# Contribution of Hypoxic Exercise Testing to Predict High-Altitude Pathology: A Systematic Review

**DOI:** 10.3390/life12030377

**Published:** 2022-03-05

**Authors:** Thomas Georges, Pierre Menu, Camille Le Blanc, Sophie Ferreol, Marc Dauty, Alban Fouasson-Chailloux

**Affiliations:** 1 CHU Nantes, Service de Médecine Physique et Réadapatation Locomotrice et Respiratoire, 44093 Nantes, France; thomas.georges@chu-nantes.fr (T.G.); pierre.menu@chu-nantes.fr (P.M.); camille.leblanc@chu-nantes.fr (C.L.B.); sophie.ferreol@chu-nantes.fr (S.F.); marc.dauty@chu-nantes.fr (M.D.); 2 CHU Nantes, Service de Médecine du Sport, 44093 Nantes, France; 3 Institut Régional de Médecine du Sport (IRMS), 44093 Nantes, France; 4 Inserm, UMR 1229, RMeS, Regenerative Medicine and Skeleton, Université de Nantes, ONIRIS, F-44042 Nantes, France

**Keywords:** hypoxia, high-altitude illness, hypoxic effort test, altitude

## Abstract

Altitude travelers are exposed to high-altitude pathologies, which can be potentially serious. Individual susceptibility varies widely and this makes it difficult to predict who will develop these complications. The assessment of physiological adaptations to exercise performed in hypoxia has been proposed to help predict altitude sickness. The purpose of this review is to evaluate the contribution of hypoxic exercise testing, achieved in normobaric conditions, in the prediction of severe high-altitude pathology. We performed a systematic review using the databases PubMed, Science Direct and Embase in October 2021 to collect studies reporting physiological adaptations under hypoxic exercise testing and its interest in predicting high-altitude pathology. Eight studies were eligible, concerning 3558 patients with a mean age of 46.9 years old, and a simulated mean altitude reaching of 5092 m. 597 patients presented an acute mountain sickness during their altitude travels. Three different protocols of hypoxic exercise testing were used. Acute mountain sickness was defined using Hackett’s score or the Lake Louise score. Ventilatory and cardiac responses to hypoxia, desaturation in hypoxia, cerebral oxygenation, core temperature, variation in body mass index and some perceived sensations were the highlighted variables associated with acute mountain sickness. A decision algorithm based on hypoxic exercise tests was proposed by one team. Hypoxic exercise testing provides promising information to help predict altitude complications. Its interest should be confirmed by different teams.

## 1. Introduction

Millions of people engage in mountain sports activities worldwide. Nepal counted nearly 1.2 million tourists in 2019, compared to 350,000 in 1995 [1]. This leads to new risks to consider. While sleeping at an altitude above 2500 m, travelers may develop high-altitude illness (HAI) due to hypobaric hypoxia [2]. The complications are acute mountain sickness (AMS), severe AMS (sAMS), high-altitude pulmonary edema (HAPE), high-altitude cerebral edema (HACE) and death. AMS occurs in approximately 10 to 25% of subjects who ascend to 2500 m after exposure to altitude for 6 to 12 h, and may concern between 50 and 85% of unacclimatized mountain travelers at 5000 m [2]. AMS is characterized by nonspecific symptoms, such as headache, nausea, vomiting, insomnia and dizziness. Some scores, such as Hackett’s score (HS), the clinical functional score, or the Lake Louise score (LLS) can help the diagnosis [3]. Severe AMS is defined by HS > 6 or LLS ≥ 6 [4]. AMS and HACE represent a continuum of the cerebral form of HAI. AMS is linked to cerebral vasodilatation secondary to hypoxemia, which induces an increase in brain volume, a decrease in compliance and an increase in intracranial pressure [5]. HACE occurs when a cytotoxic and vasogenic cerebral edema sets in [6]. HAPE represents a noncardiogenic pulmonary edema, related to a breakup of the pulmonary blood-flow barrier, responsible for an accumulation of extravascular fluid in the alveolar spaces [7]. HAPE and HACE can engage life prognosis. Prevalence of HAPE and HACE are estimated to be nearly 1% between 4000 and 5500 altitude meters [2]. The main risk factors are speed ascent and a history of such a pathology [8]. However, methods to predict those complications are lacking, particularly for travelers without previous exposure to high altitude. There is no easy way to predict how the traveler will tolerate altitude, whereas such a test would be highly beneficial. Personal physiological response to hypoxia is complex and varies widely between individuals [9]. Rest testing such as electrocardiogram or pulmonary function testing failed to predict HAI [2,10]. Some teams have reported physiological markers which could help, but with conflicting results. Oxygen saturation is easy to use in clinical practice. Karinen et al. suggested that a daily evaluation of oxygen saturation during ascent to 5300 m could help to identify a population at risk of developing AMS [11]. Conversely, Wagner et al. failed to confirm this finding. In their study, there was no difference in oxygen saturation measured at rest at 4260 m between those who experienced AMS and those who did not, when climbing to 5640 m [12]. Heart-rate variability as an index of autonomic function was also studied. Karinen et al. found that the change in heart-rate variability during ascent was related to AMS [13]. In the study by Wagner et al., the heart rate measured just before the summit was not associated to AMS [12]. Nespoulet et al. showed that a low hypoxic ventilator response measured at rest represented a predictive factor for AMS [14], which was not the case in the study by Milledge et al. [15] and those by Hohenhaus et al. [16]. Hypoxic pulmonary vascular response has also been investigated with conflicting results. Pulmonary-artery pressure measured with Doppler measurement at an FiO_2_ of 0.12 did not allow further discriminations to screen patients at risk for HAPE [16], whereas it showed an exaggerated hypoxic pulmonary vascular response in individuals susceptible to HAPE in two other studies [17,18]. However, due to a low positive predictive value, this test cannot be recommended [2]. Recently, Holmström et al. highlighted promising results concerning an association between cardiovascular response to apnea (diving bradycardia), spleen size and AMS susceptibility [19]. Hypoxic effort tests could improve the ability to detect subjects likely to develop HAI. Exercise in hypoxia, compared to rest in hypoxia, causes greater severity and incidence of acute mountain sickness [20]. Those tests aimed to evaluate physiological adaptation to hypoxia, as well as sensitivity of the chemoreceptors to hypoxic stress which are essential in adaptation to hypoxemia [21], and thus to high-altitude pathology. Worsening hypoxemia by exercise could improve the performance of those tests by challenging coping mechanisms [22,23]. These hypoxic tests are widely practiced in France, and are also prescribed for research institute members or diplomatic employees working in some countries in South America [24]. However, they have been poorly evaluated in routine practice.

The purpose of this review is to evaluate the contribution of hypoxic exercise testing, performed in normobaric conditions, to determine physiological variables associated with severe high-altitude pathologies.

## 2. Materials and Methods

### 2.1. Literature Search

We searched articles in the medical databases Pubmed, Science Direct and Embase, in October 2021. Article research extended from January 2000 to February 2022. Only studies in English language were selected. Multiples searches were carried out using the following MeSH: («Hypoxic exercise testing» OR «hypoxia exercise test») AND («High altitude illness» OR «acute mountain sickness»). The search was performed independently by two authors (AFC and TG) to assess titles and abstracts of potentially relevant articles, and then the full-text articles were retrieved. After identification of key articles, their references and citation lists were also hand-searched for further information sources. A third assessor was solicited in case of disagreement concerning an article (PM or MD).

### 2.2. Eligibility Criteria

This systematic review aimed to include research articles assessing the value of hypoxic exercise tests to determine physiological variables associated to the occurrence of severe AMS, HAPE or HACE. The effort procedure and hypoxic process had to be precisely described. It needed to concern adult people with no major medical conditions, such as respiratory or cardiac insufficiency. We selected articles assessing laboratory protocol, with tests performed before altitude exposure. We excluded field trials, where subjects were already exposed to altitude.

### 2.3. Data Extraction

All the included studies were analyzed, and data were extracted and summarized in tables using Microsoft Excel (Version 2016, Microsoft Corporation, Redmond, WA, USA): study design, year of publication, reported outcomes.

### 2.4. Quality Analysis

We used PRISMA guidelines for this review (Table S1) [25]. The included studies were appraised using GRADE approach, in order to evaluate quality of evidence [26]. This approach classifies the quality of evidence in one of four levels: high, moderate, low and very low.

## 3. Results

### 3.1. Study Selection

**Figure 1:**
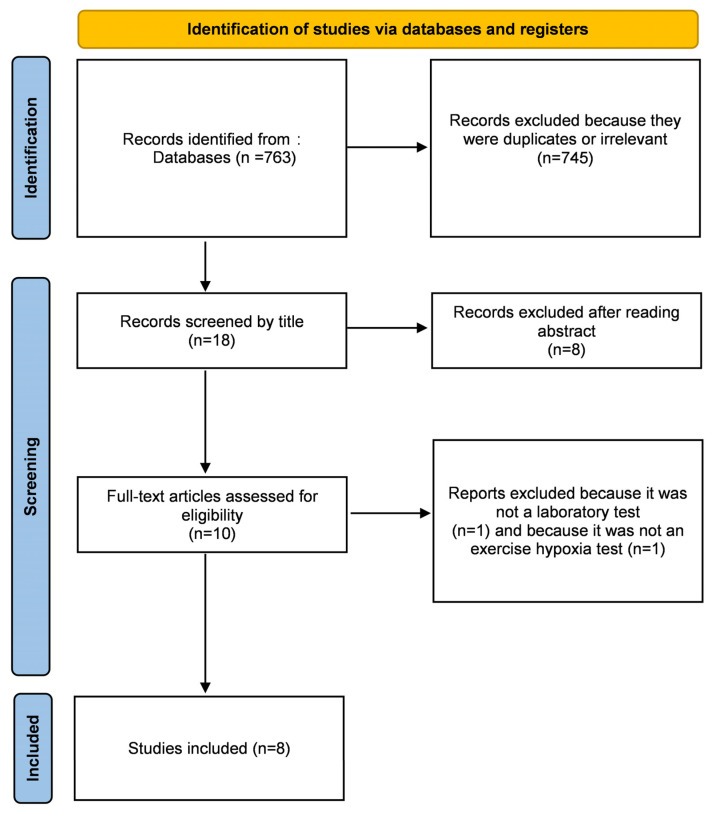
Flow chart of the included studies according to PRISMA guidelines.

Our research found 763 results. Articles were initially screened by title. After removing duplicates and reading abstracts, 10 articles were assessed for full-text reading. We excluded one of them [27] because it was not a laboratory test, and another one [17] because it was not a hypoxic exercise test (Figure 1). It was only cohort studies, as expected, for this kind of clinical question of risk evaluation [28]. All studies were monocentric, except for that of Richalet et al. [29], which included patients from distinct centers. Quality evaluation of the included studies is provided in Table 1.

There were two main statistical methods: firstly, a correlation method with the determination of a correlation coefficient [30,31]; and secondly, a measurement of the association by the determination of the odds ratio (OR) with a logistic regression, after comparison using Student t-tests for quantitative variables and Pearson’s χ^2^ test or Fisher’s exact tests for qualitative variables [29,32,33,34,35]. In one study, an analysis of variance for two factors was used [29].

### 3.2. Demographic Data

Patients’ mean age ranged from 27 to 59 years old (Table 2). Patients reached significant altitudes, ranging from 3883 to 5275 m. There was a slight masculine predominance (59%), with the exception of the study by Richalet et al. [33], which aimed to evaluate hormonal status and oral contraception on the risk of HAI. The occurrence of SHAI (24%) was concordant with the known prevalence of those pathologies [36,37] and was quite similar between studies (19–26%).

### 3.3. Diagnosis of HAI

AMS is diagnosed when typical symptoms occur after exposition at an altitude of 2500 m or higher [2]. There are no reliable measures for the diagnosis of AMS. Two main scores are used: Hackett’s score [36] and the Lake Louise Score (LLS) [38]. LLS is mainly used for research. HACE is characterized by encephalopathic symptoms, such as ataxia or decreased consciousness. It can evolve towards coma and even death [2]. HAPE manifests itself through dyspnea, cough, cyanosis and pulmonary crackles [2]. In the studies involved in this review, LLS was mainly used, except for those by Richalet et al. [32] and Canoui-Poitrine et al. [35], because they concerned a cohort that started in 1991, when LLS was not available. However, those scores have shown to be well-correlated [39]. The concept of SHAI comprises sAMS, HAPE and HACE.

AMS, HAPE and HACE often occur several hours or days after the start of altitude exposure. It is hard, in clinical routine, to evaluate the effects of hypobaric hypoxia in a large cohort of patients, hence the choice of a research team to ask their patients to fill out a questionnaire during their altitude stay and to send it back later once they returned to low altitude [29,32,33,34,35,40]. Richardson et al. [31] evaluated LLS and modified a 65-question Environmental Symptoms Questionnaire (ESQ) in normobaric conditions, right after hypoxic testing. Finally, Kammerer et al. [30] studied effects of normobaric and hypobaric hypoxia in the same patients, and filled out LLS at those particular moments, which permitted a reliable diagnosis. Subjects completed a self-reported questionnaire on a tablet. It is interesting to note that LLS was the same in hypobaric and normobaric hypoxia after exercise at 3883 m, but the low number of participants does not allow any definitive conclusion (moreover, there was no statistical analysis reported in this study).

### 3.4. Methods of Exercise Hypoxia Test

Most of these studies were published by the same team [29,32,33,34,35,40]. Thanks to its long experience in mountain medicine, the team developed a specific hypoxic exercise test, following the medical consultation. This test consisted of five 4 min phases: rest in normoxia, rest in hypoxia (by inhaling a mixture with a 0.115 fraction of inspired oxygen), exercise in hypoxia and then exercise in normoxia. The fifth phase consisted of increasing the power until the same cardiac frequency was obtained as during exercise in hypoxia. The operative power chosen for the first two exercise phases corresponded to 30% of the theoretical maximal normoxic oxygen consumption, based on heart rate. ECG was monitored, minute ventilation (VE) was measured with a metabograph and arterial saturation (SpO_2_) with a transcutaneous oximetry at an ear lobe. There were no adverse effects reported except some vagal discomfort [41].

The protocol used by Richardson et al. implied for the participants to attend the laboratory on four separate occasions, because it required an initiating session to report anthropometric measures; then three visits: one with effort in normoxia, one with hypoxia exercise at FiO_2_ 0.14 and one with hypoxia exercise at FiO_2_ 0.12. Therefore, this was more of a research protocol than a routine [31].

The protocol by Kammerer et al. consisted in 120 min of endurance exercise (including cycling and walking) at a simulated 3883 m [30]. Participants were exposed to a FiO_2_ of 0.131 in a normobaric hypoxic chamber. The material used involved a noninvasive near-infrared spectroscopy to measure cerebral oxygenation. This technology is not available in every medical center.

### 3.5. Contribution of Hypoxic Exercise Test to Predict HAI

The results of our research are provided in Table 3. Richardson et al. were able to identify several factors associated with AMS markers (LLS and change in ESQ), i.e., decrease in SaO_2_, core temperature, heart rate, physiological strain, variation in body mass index (BMI), perceived thirst, perceived exertion and perceived thermal sensation [31]. Kammerer et al. identified a new marker: cerebral oxygenation. There was a negative correlation between normobaric cerebral oxygenation decrease after exercise and LLS after 24 h in hypobaric conditions (r = −0.971; *p* < 0.01) [30]. Richalet et al. determined several physiological risk factors thanks to hypoxic exercise testing, i.e., ventilator response to hypoxia at exercise, decrease in SpO_2_ at exercise in hypoxia and cardiac response at exercise in hypoxia [32]. The last two parameters were statistically significant only in non-acetazolamide users. Adding those physiological measurements improved the discrimination between susceptible and non-susceptible subjects to HAI. They then built two risk-prediction scores (the SHAI score), according to previous exposures to altitude or not, which were a linear combination of history of severe HAI, ventilatory and cardiac response to hypoxia, desaturation during hypoxic exercise, speed of ascent, history of migraine, geographical location, female sex, age under 46 and regular physical activity. The cut-off of the score was determined at 5 for subjects with previous exposure to high altitude and at 5.5 for subjects without a previous sojourn at high altitude [35]. Next, they tested their score in some cohorts while trying to find other prognostic factors, such as blood pressure or electrocardiographic changes [34,40]. Using their protocol, they also showed that HVRe was higher in the second phase of the ovarian cycle and increased with aging [35]. To finish, they tested the robustness of their approach by setting up a prospective multicentric study to confirm the value of the SHAI score [29]. In this study, the SHAI score had a negative predictive value of 81% to detect subjects who would not suffer from SHAI.

## 4. Discussion

More and more people are faced with altitude because of the development of mountain infrastructures and air travel [1]. This phenomenon exposes such people to some serious troubles, such AMS, HAPE, HACE or even death. Simple markers to predict those events are lacking. Two important things must be considered in order to perform these diagnoses: the length of stay (HAI occurs several hours after the start of altitude exposure) and the altitude reached. Many teams attempted to find the best method to screen those patients, with conflicting results [11,12,13,14,15,16,17,18]. Apneic performance, inducing hypoxic challenge, is a promising tool [19] and needs further confirmations. Hypoxic exercise testing by increasing hypoxic stress, is also an interesting way to achieve this goal, since rest testing failed to discriminate those patients [2,10]. Interindividual variations to tolerance of hypoxia are wide, justifying personalized testing. Protocols are still few, and it is mainly the same team that have provided the most evidence on this subject [29,32,33,34,35,40].

Richalet et al. highlighted physiological risk factors for severe high-altitude illness [32]. In this study, a high desaturation in hypoxia (≥22%), a low ventilator response to exercise in hypoxia (HVRe) (<0.78 L/min/kg) and a low cardiac response to exercise in hypoxia (HCRe) (<0.84 beats/min/%) were independent risk factors for SHAI in non-acetazolamide users. Among physiological risk factors, only HVRe remained significant. The pathophysiological rationalization is interesting because, in case of lack of ventilatory or cardiac response to hypoxemia, hypoxia will be more pronounced. Hypoxia plays a crucial role in HAI, not only in sAMS and HACE but also in HAPE. Indeed, hypoxia has an action on the permeability of endothelial cells and responsible for an increase in extracellular fluid volume (vasogenic hypothesis). Moreover, hypoxia alters the efficiency of Na/K ATPase pumping, which leads in intracellular edema [6]. Finally, in HAPE, hypoxic vasoconstriction increases the vascular resistance, and thus, the risk of pulmonary edema [7].

The SpO_2_ decrease during exercise in hypoxia seems to be relevant [14,23]. Indeed, in the studies by Richardson [31], there was a correlation between the decrease in SpO_2_ and the LLQ and the ESQ, without a significant threshold being put forward. However, to date these data are still controversial [10,42,43]. Kammerer et al. did not use SpO_2_ but identified a variable which had never been described before—cerebral oxygenation (rScO_2_), with r = −0.971 (*p* < 0.01) [30]. It implies that cerebral oxygen delivery and clinical symptoms of AMS are associated, which is consistent with the pathophysiology previously mentioned.

Otherwise, Richardson et al. identified core temperature, variation of body mass index, heart rate, perceived thirst, perceived exertion and perceived thermal sensation as predictive factors, but their use in clinical practice is not easy [31]. However, the low number of included participants limits its scope considerably. Conversely, some parameters measured during the hypoxic test do not make additional contributions to the prediction risk of HAI. No ECG change and no variation in blood pressure were useful predictors of intolerance to high altitude [34,40].

Richalet et al. also studied the role of ovarian cycle in the physiologic response to hypoxia and tolerance to high altitude [33]. Indeed, sex hormones (estrogen, progesterone and testosterone) influence the control of breathing [44]. Those effects were nevertheless complex and had not been studied during exercise under hypoxic conditions. Ventilatory responses to hypoxia were higher in the luteal phase than in the follicular phase, when estrogen and progesterone were lower (0.89 ± 0.37 vs. 0.75 ± 0.27 L/min/kg (*p* = 0.03). These data were fairly consistent with previously published literature, even though the data were sparse and sometimes discordant [45,46,47,48,49]. Moreover, the practical impact of this variable remains to be discussed. Hormonal treatments and menopause status did not influence the ventilatory response to hypoxia [29].

Aging modulates the response to hypoxia. Richalet had already studied the effects of aging on physiological responses to exercise in high altitude, thanks to the same hypoxic exercise protocol. They found that cardiac response to hypoxia decreased with age, and ventilatory response to hypoxia increased with age in men, with less-pronounced desaturation in this population [50]. The reason for this increase in chemosensitivity in aging is not fully explained; the hypothesis of chronic intermittent hypoxia has been put forward [51]. In one study included in this review, an age < 46 years old was associated with an OR of 1.55 (1.01–2.37) in developing severe high-altitude pathology in non-acetazolamide users [32]. They also confirmed that HCRe decreased with age (*p* < 0.01) [33].

Frequent physical activity was associated with an OR of 1.57 to develop SHAI in non-acetazolamide users [32]. Mollard et al. already pointed out that endurance-trained women experienced more pronounced desaturation, which was linked to a reduced maximal oxygen transport with a decrement in maximal heart rate at 4500 m. In that study, the ventilatory response to hypoxia was also greater in untrained women than in trained women (0.78 ± 0.18 vs. 0.56 ± 0.20 L/min/kg, *p* < 0.05) [23]. To explain the effect of age and training in the occurrence of HAI, we can also hypothesize that younger and trained subjects are more prone to engage in vigorous activities before acclimatization, which is a risk factor for HAI.

Those studies contain some limitations. Indeed, in the study by Richardson et al. [31], in the absence of actual exposure to altitude, it seems difficult to affirm that those patients would trigger an actual HAP upon exposure to altitude. We can hardly retain the diagnosis of AMS because symptoms of HAI have been measured in normoxia just after hypoxic stress and not in hypobaric conditions during the altitude sojourn. Differences between induced hypoxemia in normobaric and hypobaric conditions have already been discussed elsewhere. It has been suggested that physiological responses, especially for ventilatory response, fluid balance and NO metabolism, are different between normobaric hypoxia and hypobaric hypoxia [52]. Physiological variables identified in this study (thermal sensation scale, decrease in body mass, perceived thirst, core temperature, rating of perceived exertion, feeling state) could be useful, but conclusions are limited due to the low number of subjects included (*n* = 12). In Kammerer’s study, the methodology also seems robust but the small number of patients (*n* = 7) makes it difficult to draw any conclusion [30].

In the studies published by Richalet et al., the two main identified biases—of which the authors are fully aware—are selection and classification biases. Indeed, the return rate of questionnaires is relatively low, and SHAI was determined via self-questionnaire [29,32,33,34,35,40]. Moreover, Bartsch et al. had already questioned the clinical relevance of those results [53]. Adding physiological variables slightly improves the performance to detect severe HAI, especially for altitude-experienced individuals [53,54]. Furthermore, until the multiSHAI study, all these studies took place in the same center. Some of the patients included may have been analyzed several times. Moreover, we can discuss the chosen hypoxic protocol. Performing the same test is probably an asset to determine a threshold and to standardize the procedure. However, we must keep in mind that this type of simulated effort (cycling) is different from the majority of the practiced activities (trekking, climbing) and that FiO_2_ is always fixed 0.115 (corresponding to an altitude of 4800 m) while travel altitudes are quite variable.

A big advantage of Richalet et al.’s approach is the very practical aspect and the presence of a clear algorithm enabling the physician facing this problem to rationalize acetazolamide prescription [29]. Moreover, this remains the largest cohort published. Given the included population and the places where these studies were carried out, it is important to remember that these results are only applicable to Western countries.

To go further and beyond the scope of this review, the possibilities of using this test and SHAI score do not stop at predicting HAI. Recently, Pla et al. conducted a study to evaluate interest of hypoxic exercise testing to predict changes in performance at moderate altitude in elite swimmers [55]. Desaturation during exercise and SHAI score correlated with a decrease in performance at altitude (1850 m).

## 5. Conclusions

The current data available about hypoxic exercise testing bring interesting physiological variables to evaluate HAP risks in healthy individuals. Nevertheless, questions arise on certain biases and cost-effectiveness of those exams. Moreover, some questions remain outstanding. The hypoxic protocol described by Richalet et al. should be extended to other teams to confirm their results. We could also consider an individualization of the hypoxic protocol by adjusting FiO2 to the aim of the traveler. Indeed, the altitude reached is the major risk factor for HAI. Thus, adapting the test could improve its performance. Finally, further studies are needed to compare hypoxic exercise testing to other hypoxic stressors, such as cardiovascular response to apnea. Those tests should help us to not forget that prevention, education and slow ascension are the keys to avoid serious trouble.

## Figures and Tables

**Table 1:** Evaluation of the level of evidence using GRADE approach.

Studies	Design	Level of Evidence	Limitations in Study Design or Execution	Inconsistency of Results	Directness	Imprecision	Quality of Evidence
Richardson et al. (2008)	Cohort study	II	-	-	Direct	+	Low
Richalet et al. (2012)	Cohort study	II	+	-	Direct	+	Moderate
Canouï-Poitrine et al. (2014)	Cohort study	II	+	-	Direct	-	Moderate
Coustet et al. (2015)	Cohort study	II	+	-	Direct	-	Moderate
Winkler et al. (2017)	Cohort study	II	+	-	Direct	-	Moderate
Kammerer et al. (2018)	Cohort study	II	-	-	Direct	+	Low
Richalet et al. (2019)	Cohort study	II	+	-	Direct	-	Moderate
Richalet et al. (2020)	Cohort study	II	+	-	Direct	-	Moderate

(+ = bias, - = no bias).

**Table 2:** Demographic data of the included studies.

Studies	Total Patients (*n*)	Mean Age (Years)	Gender (M/F)	Altitude Reached (m)	Type of Complications	Number of Complications
Richardson et al. (2008)	12	27 ± 7	12/0	NA	AMS	Not mentioned
Richalet et al. (2012)	1326	44.7 (13.8)	784/542	5079 (1038)	SHAI	318 (24%)
Canouï-Poitrine et al. (2014)	1017	44.3 (14.3)	639/378	Not mentioned	SHAI	Not mentioned
Coustet et al. (2015)	113	49.3 (14.7)	62/51	5275 (959)	SHAI	22 (19%)
Winkler et al. (2017)	182	59.3 (9.6)	Not mentioned	4861 (828)	sAMS	40 (22%)
Kammerer et al. (2018)	7	36.3 (4)	4/3	3883 (0)	AMS	Not mentioned
Richalet et al. (2019)	260	50.0 (14.9)	0/260	5011 (802)	sAMS	67 (26%)
Richalet et al. (2020)	641	50.4 (14.2)	349/292	5202 (766)	SHAI	150 (23%)
Total	3558	46.9 (13.9)	1850/1526	5092 (923)	-	597 (24%)

Abbreviations: M—male; F—Female; NA—Not applicable; AMS—Acute Mountain Sickness; SHAI—Severe High-Altitude Illness (defined as severe AMS, High-Altitude Pulmonary Edema or High-Altitude Cerebral Edema); sAMS—severe Acute Mountain Sickness. Data represent number of patients (*n*, %), mean (SD).

**Table 3:** Contribution of hypoxic exercise test to predict severe high-altitude illness.

Studies	Hypoxia Strategy	Diagnosis of HAP	Main Outcomes
Richardson et al. (2008)	125 min walking HET	LLS and ESQ measured in normobaric hypoxia	Decrease in SpO_2_ correlated to an increase in the AMS score (*p* < 0.05). Core temperature, heart rate, ‟physiological strain”, perceived thirst and decrease in body mass positively correlated (r = 0.681 and 0.667 for LLS and ESQ) to the AMS score (*p* < 0.05).
Richalet et al. (2012)	20 min HET on cycloergometer	HS, HAPE and HACE determined by questionnaire filled out by patients during their altitude stay	HVRe < 0.78 L/min/kg, ΔSae ≥ 22%, and HCRe < 0.84 beats/min/% were associated to SHAI in multivariate analysis, in NAU (OR were respectively 6.68 [3.83–11.63], 2.50 [1.52–4.11], 2.12 [1.37–2.89]). HVRe < 0.78 L/min/kg independently associated to SHAI in AU (OR 3.89 [1.74–8.73]).
Canouï-Poitrine et al. (2014)	20 min HET on cycloergometer	HS, HAPE and HACE determined by questionnaire filled out by patients during their altitude stay	HVRe, ΔSae and HCRe, AUC increased by 7% (to 0.91) in the PRE group and 17% (to 0.89) in the ABS group
Coustet et al. (2015)	20 min HET on cycloergometer	sAMS, HAPE and HACE determined by questionnaire filled out by patients during their altitude stay	No ECG characteristics predicted the risk of SHAI
Winkler et al. (2017)	20 min HET on cycloergometer	LLS, HAPE and HACE determined by questionnaire filled out by patients during their altitude stay	BP variation during HET is not a useful predictor of intolerance to high altitude
Kammerer et al. (2018)	120 min HET with alternate walking and cycling	LLS measured in hypobaric condition	rScO_2_-decrease after exercise in normobaric hypoxia correlated to AMS (r = −0.971; *p* < 0.01)
Richalet et al. (2019)	20 min HET on cycloergometer	LLS, HAPE and HACE determined by questionnaire filled out by patients during their altitude stay	HVRe was higher in the luteal phase than in the follicular phase (0.89 ± 0.37 vs. 0.75 ± 0.27 L/min/kg). Oral contraception and hormonal treatment had no effect on response to hypoxia
Richalet et al. (2020)	20 min HET on cycloergometer	LLS, HAPE and HACE determined by questionnaire filled out by patients during their altitude stay	Elaboration of a decision tree, thanks to HET, with a negative predictive value of 81% to detect subjects who will suffer from SHAI

Abbreviations: HAP—High-Altitude Pathology; SHAI—Severe High-Altitude Illness (defined as severe acute mountain sickness or high-altitude pulmonary edema or high-altitude cerebral edema); sAMS—severe Acute Mountain Sickness; AU—acetazolamide users; NAU—Non-acetazolamide users; AUC—Area Under the Curve; HVRe—ventilatory response to hypoxia during exercise; ΔSae—desaturation during exercise; HCRe = cardiac response to hypoxia during exercise; PRE—previous exposure to altitude; ABS—absence of previous exposure to altitude; HET—Hypoxia Exercise Test; BP—Blood pressure; LLS—Lake Louise score; HS—Hackett’s score; ESQ—Environmental Symptoms Questionnaire; rScO_2_—cerebral oxygenation; OR—Odds ratio.

## Data Availability

Data sharing not applicable.

## Supplementary Materials

The following are available online at https://www.mdpi.com/article/10.3390/life12030377/s1, Table S1: PRISMA 2020 checklist.
